# Apoptosis: A Comprehensive Overview of Signaling Pathways, Morphological Changes, and Physiological Significance and Therapeutic Implications

**DOI:** 10.3390/cells13221838

**Published:** 2024-11-06

**Authors:** Mohd Mustafa, Rizwan Ahmad, Irfan Qadir Tantry, Waleem Ahmad, Sana Siddiqui, Mudassir Alam, Kashif Abbas, Moinuddin, Md. Imtaiyaz Hassan, Safia Habib, Sidra Islam

**Affiliations:** 1 Department of Biochemistry, Faculty of Medicine, Jawaharlal Nehru Medical College, Aligarh Muslim University, Aligarh 202002, India; mohdmustafagc7251@gmail.com (M.M.); rizwanbiochemistry@gmail.com (R.A.); sanabiochem11@gmail.com (S.S.); moinuddin.bh@amu.ac.in (M.); 2 Department of Biochemistry, School of Biological Sciences, University of Kashmir, Srinagar 190006, India; irfanqamu@gmail.com; 3 Department of Medicine, Faculty of Medicine, Jawaharlal Nehru Medical College, Aligarh Muslim University, Aligarh 202002, India; drwaleem@gmail.com; 4 Department of Zoology, Faculty of Life Sciences, Aligarh Muslim University, Aligarh 202001, India; syedalamalig@gmail.com (M.A.); kashifabbas68@yahoo.com (K.A.); 5 Center for Interdisciplinary Research in Basic Sciences, Jamia Millia Islamia, New Delhi 110025, India; mihassan@jmi.ac.in; 6 Department of Pathology, Case Western Reserve University, Cleveland, OH 44106, USA

**Keywords:** apoptosis, signaling pathways, morphological changes, physiological significance, therapeutic strategies

## Abstract

Cell survival and death are intricately governed by apoptosis, a meticulously controlled programmed cell death. Apoptosis is vital in facilitating embryonic development and maintaining tissue homeostasis and immunological functioning. It is a complex interplay of intrinsic and extrinsic signaling pathways that ultimately converges on executing the apoptotic program. The extrinsic pathway is initiated by the binding of death ligands such as TNF-α and Fas to their respective receptors on the cell surface. In contrast, the intrinsic pathway leads to increased permeability of the outer mitochondrial membrane and the release of apoptogenic factors like cytochrome c, which is regulated by the Bcl-2 family of proteins. Once activated, these pathways lead to a cascade of biochemical events, including caspase activation, DNA fragmentation, and the dismantling of cellular components. Dysregulation of apoptosis is implicated in various disorders, such as cancer, autoimmune diseases, neurodegenerative disorders, and cardiovascular diseases. This article focuses on elucidating the molecular mechanisms underlying apoptosis regulation, to develop targeted therapeutic strategies. Modulating apoptotic pathways holds immense potential in cancer treatment, where promoting apoptosis in malignant cells could lead to tumor regression. This article demonstrates the therapeutic potential of targeting apoptosis, providing options for treating cancer and neurological illnesses. The safety and effectiveness of apoptosis-targeting drugs are being assessed in ongoing preclinical and clinical trials (phase I–III), opening the door for more effective therapeutic approaches and better patient outcomes.

## 1. Introduction

The formation and maintenance of tissues, as well as the general health of multicellular organisms, depend on the essential and highly regulated process of cell death. It orchestrates both cell proliferation and elimination, ensuring a physiological balance in adult organisms. This intricate mechanism operates during critical stages such as metamorphosis, embryogenesis, and tissue turnover and in response to pathogenic threats [1]. The two primary cell death mechanisms are programmed cell death (apoptosis) and necrosis. Apoptosis, governed by signals, involves self-destruction in response to environmental or internal cues, playing a pivotal role in maintaining tissue homeostasis by eliminating unnecessary or damaged cells. While apoptosis is tightly regulated, involving key molecules such as caspases, Bcl-2, Bax, and Bak, necrosis is a chaotic and uncontrolled form of cell destruction associated with pathological reactions following severe cellular injury [2]. Autophagic cell death and necroptosis represent additional programmed forms, contributing to the understanding of these intricate processes [3].

Apoptosis is an essential process regarding tissue homeostasis, and it eliminates superfluous cells in multicellular organisms. Several signaling pathways contribute to apoptosis, with the intrinsic (mitochondrial) and extrinsic (death receptor) pathways being the main ones. Initiator caspases (caspase-8 and -9) and executioner caspases (caspase-3, -6, and -7) play pivotal roles in apoptotic signaling cascades. The extrinsic pathway is initiated by ligands like tumor necrosis factor (TNF), forming a death-inducing signaling complex (DISC) that activates caspase-8 and downstream caspases [4,5].

The activation of intrinsic pathways in responding to various stress signals triggers mitochondrial outer membrane permeabilization (MOMP). This critical event releases various proteins into the cytosol like cytochrome c, apoptosis-inducing factor (AIF), and Smac/DIABLO. Cytochrome c activates caspase-9, while AIF contributes to caspase-independent cell death. Smac/DIABLO enhances caspase activity by blocking inhibitor of apoptosis proteins (IAPs) [6]. Understanding MOMP is crucial for unraveling disease pathogenesis and developing targeted interventions. The intrinsic pathway’s delicate balance involves pro-apoptotic and anti-apoptotic members of the Bcl-2 family. Genes such as *Fas*, *TNF Receptor 1*, *Bcl-2*, *Bid*, *Caspase-3*, *p53*, and *Cytochrome c* code for crucial proteins regulating apoptosis. Caspases, a family of proteases, play a central role in apoptosis. These proteases are categorized into initiator (caspase-8 and -9) and executioner caspases (caspase-3, -6, and -7). The Bcl-2 family of proteins, with its anti- and pro-apoptotic members, plays a crucial role in regulating apoptosis [7]. The structural arrangement of Bcl-2 with its BH domains mediates interactions between anti- and pro-apoptotic proteins, ultimately determining cell fate. The dysregulation of Bcl-2 family proteins is implicated in various diseases, making them a target for therapeutic research.

The tumor suppressor protein p53 is a crucial regulator of apoptosis and other cellular processes. Activated in response to DNA damage, p53 functions as a transcription factor, inducing pro-apoptotic genes and promoting apoptosis or cell cycle arrest. Dysfunctional or mutated p53 is common in cancers, underscoring its significance as a tumor suppressor. Cytochrome c, a heme protein essential for cellular respiration, plays a pivotal role in apoptosis by activating caspases through the formation of the apoptosome. CD95 (Fas) and TNFR1 are death receptors involved in apoptosis regulation, with CD95 promoting programmed cell death exclusively and TNFR1 having a broader role in regulating various cellular responses [8]. IAPs, a family of proteins, inhibit caspase activity, promoting cell survival by preventing caspase activation.

Apoptosis maintains overall health by eliminating cells not fit for specific functions. Understanding apoptosis’s molecular mechanisms has illuminated fundamental biological processes, paving the way for novel therapeutic strategies. Targeting apoptotic pathways is actively explored for treating diseases like cancer, neurodegenerative disorders, and autoimmune diseases [9]. Modulating apoptosis-related molecules and pathways holds potential for innovative therapies.

Efforts are required which are focused on developing anticancer agents targeting apoptotic pathways. Targeting the anti-apoptotic Bcl-2 family is a key strategy, with small interfering RNAs and microRNAs showing promise. Drugs inhibiting anti-apoptotic Bcl-2 proteins are in clinical phases, offering the potential for more effective and less toxic cancer treatments [10].

Research into apoptosis has unraveled the sophisticated molecular processes governing cell survival and death. Caspases, the chief players in apoptosis, have unveiled complex signaling pathways. Manipulating apoptosis shows promise but requires fine-tuning for specificity. Advancements in understanding apoptosis pave the way for targeted therapies, offering hope for diseases where apoptosis plays a crucial role. The prospects of apoptosis research hold promise, potentially revolutionizing health interventions. Advanced techniques in apoptosis research continue to provide insights, ensuring ongoing progress in understanding cellular biology and its implications in health and disease.

Furthermore, this comprehensive review article explores different kinds of cell death, highlighting significant targets within the apoptotic pathway for possible therapeutic interventions in human disorders. This article gives useful insights by compiling data from clinical and preclinical medical trials, paving the way for expanding our understanding of cellular death processes and their significance for medical advancements.

## 2. Primary Cell Death

**Figure 1:**
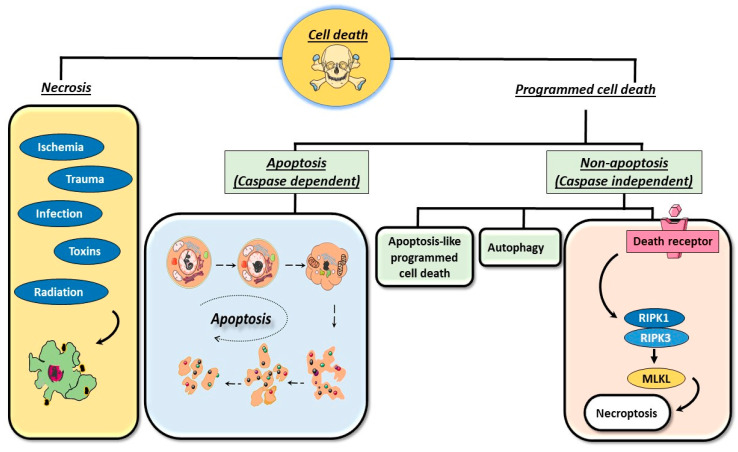
The common types of cell death: programmed cell death and necrosis. Programmed cell death includes mainly apoptosis and non-apoptotic forms of cell death, such as autophagy, necroptosis, and apoptosis-like programmed cell death. Abbreviations: RIPK1/3: receptor interacting serine threonine kinase 1/3; MLKL: mixed lineage kinase domain-like.

Cell death is a fundamental process crucial for the development, tissue maintenance, and overall health of multicellular organisms. It involves both cell proliferation and elimination to maintain physiological balance in adult organisms [11]. This process serves to remove unwanted cells during metamorphosis, embryogenesis, and tissue turnover and in response to pathogenic threats [12,13]. Two primary cell death mechanisms exist: programmed cell death and necrosis (Figure 1).

Programmed cell death, or apoptosis, is a genetically regulated process where cells undergo self-destruction in response to specific signals. These signals can be either extracellular or intracellular, directed by the cell’s environment or internal factors which include Bax, Bak, and cytochrome c [14,15]. Apoptosis plays a vital role in maintaining tissue homeostasis by eliminating unnecessary or damaged cells and regulating cell numbers in multicellular organisms [16,17]. Exogenous agents including radiation, physical trauma viruses, and bacterial toxins, and endogenous agents like TRAIL-R4 trigger programmed cell death under physiological conditions. Another group of exogenous agents are drugs like ABBV-621, MEDI3039, and Hexa-Body DR5/DR5 which are under clinical trial and can also induce apoptosis [18,19]. Additionally, internal imbalances like the withdrawal of growth factors (epidermal growth factor, nerve growth factor, etc.), loss of trophic hormone (e.g., insulin-like growth factor 1 (IGF-1) a growth hormone) stimulation [20,21], exposure to glucocorticoids, and detachment from the extracellular matrix can also induce apoptosis [22,23].

While apoptosis is often associated with programmed cell death, recent studies have shown that non-apoptotic forms of programmed cell death exist, indicating that programmed cell death and apoptosis are not synonymous [24,25]. The term “apoptosis” was first proposed by Kerr et al. (1972) to describe a distinct morphological pattern of cell death [26,27]. Apoptosis is a tightly regulated process where caspase-3, -7 and -9 act as executioners while bcl-2, Bax, and Bak play the role of regulatory molecules. The typical characteristics of apoptotic cell death are blebbing of the cell membrane, chromatin condensation, DNA fragmentation, cell shrinkage, and detachment from extracellular matrices [1,28]. Biochemically, apoptotic cell death involves the inversion of phosphatidylserine and the activation of caspases that facilitate cell death [7,29].

Apoptosis is tightly regulated while necrosis, on the contrary, is a chaotic and uncontrolled form of cell destruction. It usually involves cell enlargement, the breakdown of membranes, and the spilling of cellular material in the extracellular space. It is generally a pathological reaction following severe cellular injury. The liberated cellular material may be recognized as foreign by the immune system, resulting in an inflammatory response. Necrosis, traditionally considered a random and uncontrolled process triggered by specific stimuli like toxic trauma or physical damage, is morphologically characterized by cytoplasm and organelle swelling, plasma membrane disruption, and cell lysis [30]. Necrosis is not a completely unordered process; factors like TNFα, which are reported to be potent apoptotic inducers in a variety of cell types, can also potentially induce necrosis in the L929 murine fibrosarcoma cell line. The pan-caspase inhibitors zVAD-fmk and CrmA increase the vulnerability of L929 cells to TNFα-induced necrotic cell death. Similarly, Fas ligand causes necrosis in this cell line when zVAD-fmk is present [31]. Necrosis can lead to chronic inflammation, which may promote tumor proliferation [32]. However, recent research suggests that necrosis can also be programmed in nature. Programmed necrosis has been observed under specific conditions where apoptosis is chemically or genetically blocked or repressed [33]. Some anti-apoptotic proteins of the Bcl-2 family have been shown to inhibit both apoptotic and necrotic pathways [34]. Additionally, the depletion of ATP levels intracellularly can cause the opening of mitochondrial membrane pores, known as mitochondrial transition pores (MPTPs), which can transform an apoptotic event into a necrotic event, indicating possible interactions between these pathways [35]. However, interest in non-apoptotic forms of programmed cell death, such as autophagy, necroptosis, and apoptosis-like programmed cell death, is increasing due to the growing understanding of these processes [36,37,38,39].

Autophagic cell death, also known as type II cell death, is a self-degradative process that plays a significant role in degrading cellular components within dying cells. It is prevalent in invertebrate tissues. Necroptosis, on the other hand, is a programmed form of necrotic death, initiated by the same death signals that induce apoptosis. It occurs in response to physical traumas, infections, and various forms of neurodegeneration. It is believed that apoptosis and necroptosis share several vital processes, as certain death receptors can induce both types of cell death [40,41]. Necroptosis has characteristics of both apoptosis and necrosis. Necroptosis requires the protein RIPK3 (Receptor-interacting protein kinase-3), which was formerly known as a regulator of inflammation, cell survival, and illness, and its substrate MLKL (mixed lineage kinase domain-like pseudokinase), which is an essential participant in this process. Toll-like receptors, death receptors, interferon, etc., contribute to necroptosis [42].

## 3. Signaling Pathways of Apoptosis

Apoptosis is a highly regulated process that is essential for tissue homeostasis and eliminating unwanted or damaged cells in multicellular organisms [12]. Several signaling pathways are involved in apoptosis, with the two main pathways being the intrinsic (mitochondrial) pathway and the extrinsic (death receptor) pathway. Understanding apoptotic mechanisms is essential in comprehending the development of diseases caused by impaired apoptosis, thereby aiding the development of targeted drugs for specific apoptotic pathways or genes [43].

### 3.1. Intrinsic Pathway of Apoptosis

Intrinsic apoptosis, mediated by the mitochondrial pathway, works in a tightly regulated manner, responding to various cellular stresses that result in the release of pro-apoptotic factors. Maintaining a delicate balance between pro- and anti-apoptotic Bcl-2 family members is critical for regulating mitochondrial integrity and determining cell fate. The intrinsic pathway culminates in the dismantling of the cell, with apoptotic cells undergoing changes in their cell surface, facilitating recognition and engulfment by phagocytes [44].

#### 3.1.1. Mitogen-Activated Protein Kinase (MAPK) Signaling

Mitogen-activated protein kinase (MAPK) signaling plays a complex and context-dependent role in the regulation of apoptosis, influencing both pro-apoptotic and anti-apoptotic processes. The MAPK pathway is a conserved intracellular signaling cascade that responds to various extracellular stimuli, including growth factors, stress signals, and cytokines. Three major MAPK subfamilies involved in apoptosis regulation are the Extracellular Signal-Regulated Kinases (ERKs), c-Jun N-terminal Kinases (JNKs), and p38 MAPK [45]. In certain contexts, the activation of ERKs promotes cell survival and inhibits apoptosis [45]. This can occur through the upregulation of anti-apoptotic proteins, such as members of the Bcl-2 family, or the inhibition of pro-apoptotic factors. JNK signaling can either promote cell survival or induce apoptosis, depending on the cellular context and stimuli. Activation of JNKs is often associated with stress-induced apoptosis. JNKs can phosphorylate and activate pro-apoptotic Bcl-2 family members, disrupt mitochondrial membrane integrity, and stimulate the release of pro-apoptotic factors [45,46].

Stress Response: Similar to JNKs, p38 MAPK is activated by cellular stress and plays a role in stress-induced apoptosis. It can modulate apoptosis by influencing the expression of pro-apoptotic and anti-apoptotic proteins, as well as by regulating the activity of transcription factors involved in apoptosis [47].

The MAPK pathways are interconnected, and cross-talk between them adds another layer of complexity to apoptosis regulation. For example, ERK activation may inhibit JNK-induced apoptosis, highlighting the intricate balance between survival and death signals [47].

**Figure 2:**
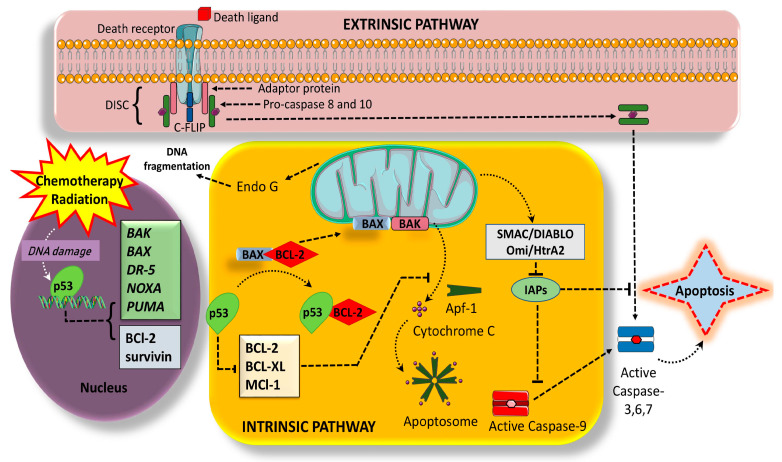
The intrinsic and extrinsic pathways of apoptosis. Intrinsic pathway is activated by various intracellular signals, like hypoxia, DNA damage, oxidative stress, etc., leading to mitochondrial dysfunction and release of pro-apoptotic factors like cytochrome c leading to activation of initiator caspase 9. The extrinsic pathway is activated by TNFα/Fas ligands which bind to their respective receptors on the cell membrane, leading to activation of initiator caspase 8. Initiator caspases (caspase 8, 9, 10) are pivotal in both pathways and catalyze the proteolytic maturation of effector caspases (such as caspase 3, 6, 7), leading to the initiation of a caspase cascade, ultimately resulting in cell demolition. The p53 tumor suppressor protein induces transcription of various pro-apoptotic genes like *BAX*, *BAK*, *DR-5*, *NOXA*, and *PUMA* and inhibits transcription of the anti-apoptotic gene *BCL-2*. This intricate network of interactions eventually triggers the apoptotic process. Abbreviations: BAX: Bcl-2-associated protein x; BAK: Bcl-2 homologs antagonist/killer; DR-5: death receptor 5; *NOXA*: pro-apoptotic member of BH3 only protein; *PUMA*: p53 upregulated modulator of apoptosis; p53: tumor protein P53.

Regulation of MAPK Activity: Feedback mechanisms exist to regulate the duration and amplitude of MAPK signaling [48]. These mechanisms involve phosphatases and negative regulators that ensure a controlled response to stimuli, preventing excessive or prolonged activation that could lead to apoptosis. MAPKs are involved in the transduction of signals from death receptors, such as Fas and TNF receptors, leading to apoptosis. This involves the activation of downstream caspases and the regulation of pro-apoptotic Bcl-2 family members (Figure 2). The role of MAPK signaling in apoptosis is highly nuanced, with context-dependent effects influenced by the specific stimuli, cellular conditions, and the intricate interplay with other signaling pathways. While MAPKs can promote cell survival, they also contribute to apoptotic processes, reflecting the complex regulatory networks that govern cellular fate decisions [49].

#### 3.1.2. JAK-STAT Signaling

The activation of the JAK-STAT pathway occurs upon ligand binding, which includes cytokines, growth factors, or hormones, to cell surface receptors. These receptors are associated with Janus kinases (JAKs), which become active upon ligand binding. This activation leads to conformational changes in the receptor, subsequently activating JAKs associated with it. Phosphorylation of tyrosine residues on the receptor creates docking sites for Signal Transducer and Activator of Transcription (STAT) proteins. Once recruited, STAT proteins undergo phosphorylation by JAKs, forming dimers that translocate to the nucleus. Within the nucleus, STAT dimers bind specific DNA sequences, regulating the transcription of target genes involved in cell survival, proliferation, differentiation, and apoptosis [50].

The JAK-STAT pathway exhibits dual effects on apoptosis, with some STAT-regulated genes, including Bcl-2 family members, inhibiting apoptosis by preventing the release of mitochondrial cytochrome c. Conversely, other target genes may promote apoptosis by activating pro-apoptotic pathways. The delicate balance between anti-apoptotic and pro-apoptotic signals is pivotal in determining cell fate. Dysregulation of the JAK-STAT pathway is implicated in diseases like cancer, where aberrant survival signaling can hinder apoptosis [51].

STAT3 is a transcription factor crucial in the JAK-STAT pathway, participating in cellular processes like survival, proliferation, and differentiation. STAT3 primarily exerts an anti-apoptotic function by upregulating anti-apoptotic genes, such as Bcl-2 and Bcl-xL. These proteins inhibit the intrinsic apoptotic pathway by preventing the release of cytochrome c from mitochondria. Moreover, STAT3 can directly or indirectly inhibit pro-apoptotic factors, suppressing the transcription of pro-apoptotic Bcl-2 family members or inhibiting caspase activation [52].

STAT3 also contributes to mitochondrial protection, crucial in averting apoptosis initiation. It interacts with various signaling pathways, such as PI3K-Akt, indirectly influencing apoptosis. Aberrant activation of STAT3 is common in cancer, promoting cell survival and resistance to apoptosis. The role of STAT3 in apoptosis is context-dependent, as it can either promote cell survival or contribute to pro-apoptotic signals based on specific conditions or cell types [53].

As STAT3 is recognized as a potential therapeutic target in cancer, the inhibition of its signaling is explored to induce apoptosis and overcome resistance to cell death. However, the multifunctional nature of STAT3, its context-dependent effects, and its interactions with other pathways highlight the complexity of its role in apoptosis. Understanding these mechanisms is crucial for developing targeted therapies, particularly in diseases characterized by disrupted cell survival like cancer [54]. Moreover, the STAT pathway contributes to the induction of NF-κB [55]. Activation of the tripartite IKK complex induces phosphorylation-mediated degradation of IκB, primarily relying on IKKβ activity [56]. This pathway governs both cell survival and death, with the anti-apoptotic role of the IKKβ-driven classical pathway being crucial, especially in the modulation of various immunoreceptors [57].

Apart from extrinsic and intrinsic pathways, there are less well-known pathways of caspase activation, including the involvement of caspase-12 or caspase-2, which is an enzyme that participates in the stress-induced apoptosis pathway of the endoplasmic reticulum (ER). Caspase-12 is found in rodents (and its human counterpart caspase-4 in ER-stress-induced apoptosis), and its principal function is to mediate apoptosis in response to stress conditions within the ER. The endoplasmic reticulum is essential in protein folding and processing, and an accumulation of unfolded or misfolded proteins (ER stress) causes a cellular reaction known as the unfolded protein response (UPR). This can promote the release of pro-inflammatory cytokines, therefore coupling ER-stress-induced apoptosis to inflammatory reactions. Dysregulation of caspase-12 has been linked to chronic illnesses such as cancer [31]. The other less known pathway is the perforin/granzyme pathway, which induces apoptosis through granzyme A or granzyme B. Granzyme A and granzyme B both trigger apoptosis in target cells, although by distinct mechanisms. GrB promotes caspase-dependent apoptosis, whereas GrA triggers apoptosis via caspase-independent pathways. Depending on the environment and the kind of target cells confronted by cytotoxic lymphocytes, the specific granzyme(s) engaged in the immune response may differ [58,59]. Granzyme A cleaves and activates specific substrates, such as nucleoplasmin, resulting in chromatin condensation and death outside the normal caspase-dependent mechanism. GrB, on the other hand, largely promotes the caspase-dependent apoptotic pathway by cleaving cellular substrates such as caspase-3, caspase-7, and the pro-apoptotic protein Bid [60]. These apoptotic pathways ultimately converge on the same terminal or execution pathway [61]. Key regulatory proteins in both intrinsic and extrinsic pathways are the caspases. Mammalian caspases are categorized into three groups based on their specific functions: developmental, inflammatory, and apoptotic pathways [62,63]. Caspases can be classified as initiator (caspase-8 and -9) and effector or executioner caspases (caspase-3, -6, and -7), depending on their position in apoptotic signaling cascades [64,65,66]. Initiator caspases are activated by auto-cleavage and subsequently cleave and activate downstream “executioner” caspases, leading to the proteolytic dismantling of the cell [67,68,69].

BH3-only proteins (*BAX* and *BAK*) become activated following apoptotic stimuli and trigger the release of cytochrome c and Smac/DIABLO, forming the apoptosome complex [70,71,72,73]. The apoptosome activates the initiator caspase-9, which, in turn, triggers the caspase-3 cascade, eventually causing cell demolition [74].

AIF also plays a role in cell survival. Smac/DIABLO and Omi/HtrA2, other released mitochondrial proteins, interact with inhibitor of apoptosis (IAP) proteins, promoting caspase activation [75,76,77]. In summary, understanding apoptotic pathways is vital for unraveling disease pathogenesis and advancing drug development targeting specific apoptotic mechanisms.

#### 3.1.3. Mitochondria-Dependent Cell Death: The Role of MOMPs

The intrinsic (mitochondrial) pathway is activated by various stress signals, such as reactive oxygen species (ROS), DNA damage, endoplasmic reticulum stress, damaged cytoskeleton, loss of cell adhesion, withdrawal of growth factors, and macromolecular synthesis inhibition, among others [78,79,80,81,82]. Key features of apoptosis, such as fragmentation of DNA, condensation of chromatin, loss of plasma membrane asymmetry, and blebbing of cells, rely on the activation of caspases [83,84,85,86]. These caspases play a critical role in cleaving specific cellular substrates [87].

**Figure 3:**
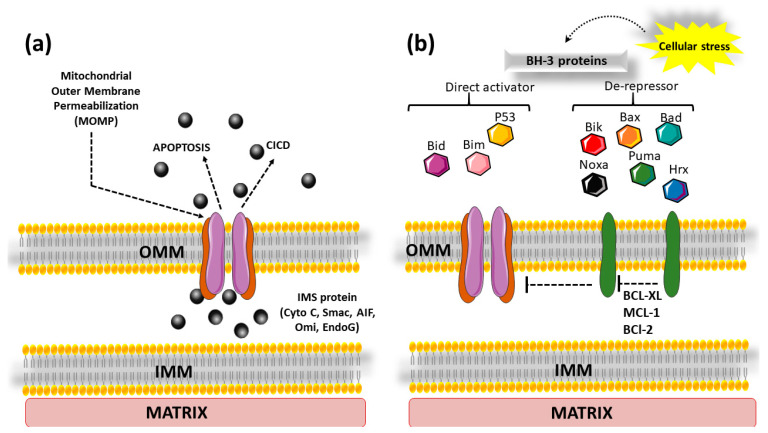
Role of mitochondrial outer membrane pores (MOMPs) in apoptosis. (**a**) Various internal stresses lead to MOMP formation, which leads to the release of cytochrome c into the cytoplasm, resulting in the activation of caspases and finally culminating in apoptosis or cell death through a caspase-independent manner (CICD); (**b**) cellular stress activates BH3 proteins (Bim, Bid) and p53 which either directly activate BAX or BAK to form pores in the OMM and finally result in apoptosis or inhibit anti-apoptotic proteins (BCL-2, BCL-xL and MCL-1) by de-repressor BH3-only proteins (like Bik, Noxa, Puma, etc.). Abbreviations: BCL-2: B-cell leukemia/lymphoma 2 protein; BCL-Xl: B-cell lymphoma extra-large; BAX: Bcl-2-associated protein x; BAK: Bcl-2 homologs antagonist/killer; Bid: BH3-interacting domain death agonist; Bim: Bcl-2-interacting mediator of cell death; Bik: BCL-2-interacting killer; MCL-1: myeloid cell leukemia sequence 1; *NOXA*: pro-apoptotic member of BH3 only protein; OMM: outer mitochondrial membrane; IMM: inner mitochondrial membrane; IMS: intermembrane space; *PUMA*: p53 upregulated modulator of apoptosis; p53: tumor protein P53.

In the mitochondrial pathway, the activation of caspases hinges on MOMP, a crucial event often referred to as the ‘point of no return’ during apoptosis [87]. This event releases various proteins into the cytosol (Figure 3a). One such protein is cytochrome c, which binds to apoptotic protease-activating factor-1 (APAF-1), triggering the formation of the apoptosome [88]. Subsequently, this leads to the activation of initiator caspase-9 and executioner caspase-3, ultimately culminating in cell death. Apoptosis-inducing factor (AIF) is another example of a protein that is released by the mitochondria [89]. In contrast to cytochrome c, AIF migrates into the nucleus, where it contributes to chromatin condensation and DNA breakage, hence facilitating caspase-independent cell death mechanisms. Smac/DIABLO, another protein generated during MOMP, is a pro-apoptotic protein that works by blocking inhibitor of apoptosis proteins (IAPs), enabling caspases to become more efficient in promoting apoptosis [90]. Smac interacts with IAPs, specifically targeting their baculovirus IAP repeat (BIR) domains. IAPs, which include proteins like XIAP (X-linked inhibitor of apoptosis protein), inhibit caspase activity by binding to IAPs [91].

Besides cytochrome c, AIF, and Smac/DIABLO, mitochondria also release endonuclease G and Omi/HtrA2. Endonuclease G participates in DNA fragmentation during apoptosis. Omi/HtrA2 is also a pro-apoptotic molecule that works similarly to Smac/DIABLO. Figure 3b describes the role of cellular stress that initiates the activation of pro-apoptotic BH-3 proteins. Bax, Bid, and various other proteins govern cellular fate via regulating apoptosis, which is required for the health of tissue. The dynamic interaction of pro-apoptotic and anti-apoptotic components of the Bcl-2 family determines whether cells survive or die, which has implications for cancer research and therapeutic therapies targeting apoptotic pathways. This topic is explained in detail under “The Bcl-2 family of proteins”.

#### 3.1.4. Cytosolic Cytochrome c: A Potent Pro-Apoptotic Protein

**Figure 4:**
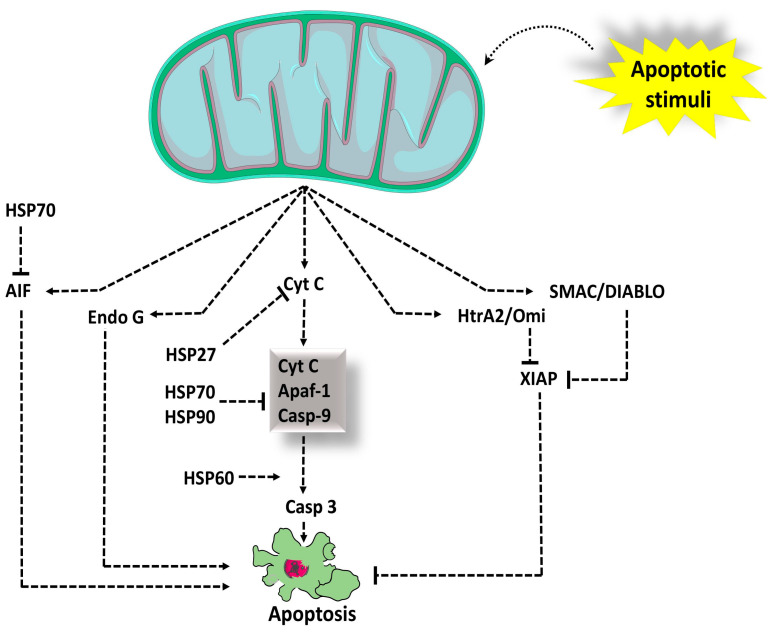
Apoptotic stimuli activate mitochondria to release Cyt c into the cytoplasm. Cyt c, once in the cytoplasm, binds with Apaf-1 and procaspase-9 to form a complex called the apoptosome. The apoptosome leads to the activation of procaspase-9 to caspase-9 which leads to activation of executioner caspase-3 which favors apoptotic cell death. HSP at different levels controls the apoptotic cascade right from Cyt c to caspase-3. Smac/Diablo and HtrA2 negatively regulate XIAP. AIF and Endo-G, once released, cause chromatin condensation and DNA fragmentation (hallmarks of apoptosis) in a caspase-independent fashion. Abbreviations: AIF: apoptosis-inducing factor; Apaf: apoptotic protein-activating factor; casp-9, caspase-9; Cyt.c, cytochrome c; Endo-G: endonuclease-G; HSP: heat shock protein; HtrA2/Omi: high-temperature requirement protein A2; Smac/DIABLO: Second mitochondria-derived activator of caspases/Direct Inhibitor of Apoptosis-Binding protein with a low isoelectric point; XIAP: X-linked inhibitor of apoptosis protein.

The mitochondrial intermembrane space (IMS) houses a diverse group of proteins that play a critical role in promoting cell death upon their release. Among these pro-apoptotic proteins, Cytochrome c (Cyt c) stands out as the first identified component at the molecular level. These events are meticulously regulated by various heat shock proteins (HSPs) (Figure 4) and eventually enable the catalytic maturation of caspase-3 and other caspases, ultimately orchestrating the biochemical and morphological characteristics of apoptosis [92,93,94]. Furthermore, other soluble proteins released from mitochondria during apoptosis induction contribute to the caspase cascade by counteracting caspase-inhibitory proteins, IAPs [95]. One notable example is the Second mitochondria-derived activator of caspases/Direct Inhibitor of Apoptosis-Binding protein with a low isoelectric point (Smac/DIABLO), which exposes its N-terminus through proteolytic maturation when the precursor polypeptide is imported into the mitochondria [96]. This exposed N-terminus can interact with and neutralize IAPs when the protein is released into the cytosol. Similarly, the high-temperature requirement protein A2 (HtrA2, also known as Omi), a serine protease, contains an IAP-binding N-terminus, contributing to the regulation of apoptosis [97].

Apoptosome formation is a crucial step in the intrinsic or mitochondrial pathway of apoptosis, which is a programmed cell death process. A more detailed explanation of the process is provided as follows:

In response to various cellular stress signals, such as DNA damage or cellular injury, mitochondria release cytochrome c into the cytoplasm [98]. Once in the cytoplasm, cytochrome c binds to Apaf-1, which is a cytosolic protein known as apoptotic protease-activating factor 1 [99]. The binding of cytochrome c induces a conformational change in Apaf-1, allowing it to form a large oligomeric structure called the apoptosome [100]. Apaf-1 oligomerization leads to the recruitment and activation of procaspase-9 molecules. Procaspase-9 is an inactive form of caspase-9, which is a key enzyme involved in initiating the apoptotic cascade [101]. Within the apoptosome, the procaspase-9 molecules undergo auto-activation, where one procaspase-9 molecule cleaves another to generate active caspase-9 [102]. This active caspase-9 then triggers a cascade of downstream events by activating other caspases, such as caspase-3 and caspase-7, which ultimately lead to the execution of apoptosis [103]. The formation of the apoptosome is a critical event in the intrinsic pathway of apoptosis, which is regulated by the balance of pro-apoptotic and anti-apoptotic proteins, particularly in the mitochondria. This pathway plays a crucial role in normal development, tissue homeostasis, and the elimination of damaged or unwanted cells [104].

### 3.2. Extrinsic (Death Receptor) Pathway

The extrinsic pathway is initiated by extracellular death ligands binding to specific death receptors on the cell surface. The binding of ligands, such as tumor necrosis factor (TNF) and Fas ligand (FasL), to their respective receptors triggers the following steps:

#### 3.2.1. Death Receptor Activation

Death receptor activation is a key process in the initiation of apoptosis, or programmed cell death. Death receptors are a class of cell surface receptors that include TNF receptor 1 (TNFR1) and Fas (CD95) [8,105,106,107]. When a ligand, such as tumor necrosis factor (TNF) or Fas ligand (FasL), binds to the death receptor on the cell surface, it induces the trimerization of the death receptor. The trimerization of death receptors enables the recruitment of adaptor proteins to the intracellular side of the receptor. Two important adaptor proteins involved in death receptor signaling are the Fas-associated death domain (FADD) and TNF receptor-associated death domain (TRADD) [108]. FADD contains a death domain that interacts with the death domain of the death receptor. TRADD also contains a death domain and can be associated with the death receptor of TNF through this domain [109,110]. These interactions between the adaptor proteins and death receptors are crucial for transmitting apoptotic signals. Once FADD or TRADD is recruited to the death receptor complex, it can further recruit and activate downstream signaling molecules, forming a complex called the death-inducing signaling complex (DISC). The DISC activation leads to the activation of caspases, which are enzymes that play a central role in the execution of apoptosis [4,111,112,113,114]. Overall, death receptor activation triggers a series of molecular events that ultimately result in programmed cell death. This process is important for regulating various physiological and pathological processes, including immune response, tissue homeostasis, and elimination of damaged or infected cells [115,116,117,118].

#### 3.2.2. Caspase-8 Activation

The attachment of FADD to the death receptor recruits procaspase-8, resulting in its activation and the formation of the death-inducing signaling complex (DISC). Alternatively, TRADD can recruit receptor-interacting protein kinase 1 (*RIPK1*), leading to the formation of the ripoptosome complex. Cross-talk between RIPK1 and TRADD is required for TNF signal transmission [119]. The ripoptosome is a signaling complex that plays a role in necroptosis. This activates caspase 8, which is generally linked with apoptosis, but in the setting of ripoptosome stimulation, it helps facilitate cell death pathways, connecting the necroptosis-apoptosis pathways. Activated caspase-8 then directly activates downstream effector caspases, such as caspase-3 and caspase-7 [120,121,122].

### 3.3. The Regulatory Role of Genes and Their Protein Products, and the Convergence of Intrinsic and Extrinsic Pathways

Caspase-8 can also cleave and activate the Bid, which is a member of the pro-apoptotic Bcl-2 family, leading to the generation of its truncated form known as tBid. This tBid can translocate to the mitochondria and induce MOMP, initiating the intrinsic pathway [123]. It is important to note that the intrinsic and extrinsic pathways are interconnected and can cross-regulate each other at various points, amplifying the apoptotic signal. Additionally, other pathways, such as the endoplasmic reticulum stress pathway, can also contribute to apoptosis under specific conditions [124,125].

#### 3.3.1. Genes and the Translation Products Involved in Apoptosis

The intrinsic pathway is regulated by a delicate balance between pro-apoptotic and anti-apoptotic members of the Bcl-2 family of proteins; it is a highly regulated process essential for maintaining tissue homeostasis, eliminating damaged or infected cells, and preventing the proliferation of potentially harmful cells. A few genes that code for such proteins are Fas (*FAS*), TNF Receptor 1 (*TNFRSF1A*), Bcl-2 (*BCL2*), Bid (*BID*), Caspase-3 (*CASP3*), p53 (*TP53*), Cytochrome c (*CYCS*), etc. Several molecules and proteins play crucial roles in orchestrating the apoptotic process. In this review, we discuss some of the key players in detail:

#### 3.3.2. Caspases

Caspases are a family of proteases (enzymes that cleave proteins) that play a central role in apoptosis. They exist in an inactive form and are activated during apoptosis to execute the dismantling of the cell. There are two main types of caspases in apoptosis: initiator caspases (e.g., caspase-8 and caspase-9) and executioner caspases (e.g., caspase-3 and caspase-7). Initiator caspases activate executioner caspases, leading to the degradation of cellular components and eventual cell death [126,127,128,129].

#### 3.3.3. Bcl-2 Family of Proteins

The Bcl-2 family of proteins plays a crucial role in regulating apoptosis. The Bcl-2 homology (BH) domains are an essential component of the conserved structure seen in the Bcl-2 protein family. This family comprises both anti- and pro-apoptotic proteins. The anti-apoptotic members, such as Bcl-2, have four BH domains (BH1 to BH4) that result in the formation of a globular structure. Contacts with pro-apoptotic proteins are mediated by BH1 to BH3 domains, and membrane adherence is regulated by BH4. This structural arrangement regulates apoptosis [130]. This family includes both pro- and anti-apoptotic members, and their interactions ultimately determine cells’ fate. Pro-apoptotic members, such as Bax and Bak, promote apoptosis by inducing the release of cytochrome c from the mitochondria, which triggers a cascade of events leading to cell death. These proteins form pores in the outer mitochondrial membrane, facilitating the release of pro-apoptotic factors into the cytosol. On the other hand, anti-apoptotic members, like Bcl-2 and Bcl-xL, function to prevent apoptosis and promote cell survival [131]. They do so by binding and sequestering the pro-apoptotic Bax and Bak proteins, preventing them from forming pores in the mitochondrial membrane and inhibiting the release of cytochrome c. The balance between pro- and anti-apoptotic is crucial. When the pro-apoptotic signals outweigh the anti-apoptotic signals, the cell is more likely to undergo apoptosis. Conversely, when the anti-apoptotic signals dominate, the cell is more likely to survive and continue its normal functions. Dysregulation of Bcl-2 family proteins can have significant implications in various diseases, including cancer and neurodegenerative disorders, where an abnormal balance between pro- and anti-apoptotic factors can contribute to uncontrolled cell growth or cell death [132,133]. As a result, Bcl-2 family proteins have been a target of intense research, to develop therapies that can selectively modulate apoptosis to treat different types of malignancies [134].

#### 3.3.4. Tumor Suppressor Protein p53

The tumor suppressor protein p53 is indeed a crucial regulator of apoptosis, as well as other cellular processes [135]. It plays a central role in maintaining genomic stability and preventing the formation of cancer. When a cell undergoes DNA damage or other cellular stresses, p53 is activated and accumulates in the nucleus [136]. This activation can occur through various mechanisms, such as post-translational modifications or stabilization of the protein. Once activated, p53 functions as a transcription factor, binding to specific DNA sequences near target genes and promoting their transcription [137,138]. One of the main outcomes of p53 activation is the induction of pro-apoptotic genes [139]. These genes encode proteins that promote cell death, triggering the process of apoptosis. By promoting apoptosis in damaged or stressed cells, p53 helps eliminate potentially harmful cells from the body, reducing the risk of cancer development [139,140]. Additionally, p53 can also induce cell cycle arrest, allowing time for DNA repair before the cell proceeds through the cell cycle. p53 is a critical guardian of the genome, responding to cellular stress by either promoting apoptosis or halting the cell cycle for repair [141,142]. Dysfunctional or mutated p53 is commonly found in various cancers, highlighting its significance as a tumor suppressor [143,144,145]. Understanding the mechanisms of p53 regulation and function is essential for developing targeted cancer therapies and advancing our knowledge of cell biology and cancer biology [146,147,148].

The p53 protein, often referred to as the “guardian of the genome”, is a tumor suppressor protein that plays a crucial role in cell division regulation and preventing tumor formation. It is encoded by the *TP53* gene and is one of the most important tumor suppressors in the human body [149]. The mechanism of p53 involves its activation, DNA binding, and subsequent regulation of target genes [150]. The activity of p53 is tightly regulated under normal conditions. It is mainly regulated by its interaction with a negative regulator called MDM2 (Mouse Double Minute 2). MDM2 binds to p53 and marks it for degradation, keeping its levels low in the cell [151]. However, various cellular stresses such as DNA damage, hypoxia, or oncogene activation can lead to the activation of p53 [3]. Upon activation, p53 undergoes a series of modifications, including phosphorylation, acetylation, and other post-translational modifications, which stabilize and activate the protein. Once activated, p53 acts as a transcription factor, meaning it can bind directly to specific DNA sequences in the promoter regions of its target genes. Once bound to DNA, p53 regulates the expression of a wide array of target genes involved in various cellular processes, including DNA repair, cell cycle arrest, apoptosis, and senescence (cellular aging). The specific response triggered by p53 depends on the nature and extent of cellular damage [152].

In response to DNA damage, p53 induces the expression of genes like p21 (CDKN1A), which halts the cell cycle at the G1/S or G2/M checkpoints. This arrest provides time for DNA repair before cell division. p53 activates the expression of genes involved in DNA repair mechanisms, enhancing the cell’s ability to fix damaged DNA and maintain genomic stability [153]. If the DNA damage is extensive and irreparable, p53 promotes the expression of pro-apoptotic genes like *BAX*, *PUMA*, and *NOXA*, leading to programmed cell death. This prevents the propagation of cells with severe DNA damage that could potentially develop into cancer. In certain circumstances, p53 induces cellular senescence, a state of irreversible growth arrest that prevents damaged cells from proliferating. Overall, the proper functioning of p53 is vital for maintaining genome stability and preventing the development of cancer. Mutations in the *TP53* gene can lead to dysfunctional p53, which compromises its tumor-suppressing capabilities, contributing to the uncontrolled growth of cancer cells [154,155].

#### 3.3.5. Calpains and Calcium Signaling by IP_3_ Receptor

Calcium-independent proteases, known as caspases, have an established role in apoptosis. Apart from this, non-caspase proteases like calpains, cathepsins, granzymes, and the proteasome also execute apoptosis [156]. The calpains are broadly classified as tissue-specific calpains and ubiquitous calpains. The tissue-specific group includes skeletal-muscle-specific calpain-3 and stomach-specific calpain-9, for example. Calpain I and calpain II are among the ubiquitous calpains. They are identified by their requirement for different in vitro concentrations of Ca^2+^ for activation. A concentration of 2–80 µM is required for calpain I, and a concentration of 0.2–0.8 mM of Ca^2+^ for calpain II [157,158]. The calpain family is conserved from fungi to humans [159]. Typical calpains (1, 2, 3, 8, 9, 11, 12, and 14) contain a penta-EF hand Ca^2+^ in domain IV that can bind the calpain small subunit (only calpains 1, 2, and 9 have been shown to dimerize) or calpastatin [160,161]. Atypical calpains (5, 6, 7, 10, 13, and 15) lack a penta-EF hand in domain IV and are unable to bind the calpain small subunit or calpastatin. The cytosolic Ca^2+^ concentration in resting cells is ~100 nM and is maintained by enzymes that translocate Ca^2+^ ions across the plasma membrane or into intracellular stores. Mitochondria can modulate the kinetics and cellular distribution of Ca^2+^ ions and signals by sequestering Ca^2+^ ions. This Ca^2+^ is utilized for the activation of matrix enzymes for ATP production [162,163].

Cytosolic Ca^2+^ levels are increased physiologically when cells are challenged with fluctuating levels of hormones and growth factors or mechanical deformation. The Ca^2+^ ions can be released from intracellular stores and then enter the cell across the plasma membrane. For mobilizing Ca^2+^ stores, inositol 1,4,5-trisphosphate (IP_3_) receptors are a principal route in most of the cells. The release of Ca^2+^ via IP_3_ receptors stimulates diverse cellular activities that are vital and critical for life, such as post-fertilization Ca^2+^ oscillations. However, under some conditions, IP_3_-receptor-mediated Ca^2+^ signals destabilize the cell, resulting in cellular demise [164,165,166].

IP_3_ receptors are large (~1200 kDa) tetrameric proteins. The subunit of each projects an amino-terminal domain into the cytoplasm. Membrane-spanning carboxy-terminal regions form an integral Ca^2+^ channel [167]. IP_3_ binding by the amino-terminal domains causes a conformational change that promotes channel opening. The IP_3_ binding site and transmembrane regions contain a regulatory area. Although IP_3_ is necessary to activate IP_3_ receptors, the activation of these channels also depends on the ambient Ca^2+^ concentration. At a concentration of approximately 500 nM, Ca^2+^ works synergistically with IP_3_ to activate IP_3_ receptors. However, at higher concentrations, cytosolic Ca^2+^ inhibits IP_3_ receptor regulatory and activity sites. The inhibition of IP_3_ receptors by Ca^2+^ is thought to be a crucial mechanism and a druggable target for terminating channel activity and thus preventing pathological Ca^2+^ rises [168,169].

#### 3.3.6. Cytochrome c

Cytochrome c is a heme protein that is essential for cellular respiration. It is located in the IMM and is involved in electron transfer between the ETC complexes III and IV—the heme group is a prosthetic group comprising a Ferrous atom coordinated with a porphyrin ring. The heme group is buried inside protein and is involved in accepting and transferring electrons because iron can exist in different oxidation states. The general structure of cytochrome c is conserved across species, indicating its importance in cellular respiration. Upon binding to the Apaf-1 protein in the cytoplasm, it forms the apoptosome, which activates initiator caspases, resulting in the activation of an apoptotic cascade [170,171].

#### 3.3.7. Death Receptors

Although both the CD95 (Fas) receptor and the TNFR1 (Tumor Necrosis Factor Receptor 1) are involved in regulation through apoptosis, they differ in their functionality. CD95 possesses an internal death domain that is essential for apoptosis signaling, whereas TNFR1 comprises extracellular cysteine-rich regions for ligand binding and a death domain that regulates a variety of cellular responses, including inflammation and apoptosis. CD95 promotes programmed cell death (apoptosis) exclusively, whereas TNFR1 has a broader role, regulating inflammation and immunological responses in addition to apoptosis [171].

### 3.4. Inhibitor of Apoptosis Proteins (IAPs)

IAPs are a family of proteins that can inhibit caspase activity and promote cell survival. They can block apoptosis by binding to and inhibiting caspases, thus preventing their activation [172,173,174]. These players form a complex network of interactions and cross-talk to determine whether a cell will undergo apoptosis or evade its fate. Apoptosis can lead to severe consequences if it becomes out of control (dysregulated) and can contribute to various diseases, like cancer, neurodegenerative disorders, and autoimmune diseases. Researchers continue to study these key players and their regulatory networks to gain a deeper understanding of apoptosis and develop potential therapeutic interventions.

## 4. Role in Health and Disease

Apoptosis balances the overall health of an individual by eliminating cells that are not in a condition to perform their specific function. Dysregulation of apoptosis has been implicated in a wide range of diseases, including cancer, neurodegenerative disorders, autoimmune diseases, and cardiovascular diseases [175,176,177,178,179,180]. Understanding the molecular mechanisms underlying apoptosis has not only shed light on fundamental biological processes but also paved the way for the development of novel therapeutic strategies [181]. The intricate understanding of apoptosis has opened new avenues for therapeutic interventions. Targeting apoptotic pathways holds immense potential for the treatment of various diseases. Strategies aimed at inducing apoptosis in cancer cells or preventing excessive cell death in neurodegenerative disorders are actively being explored. Furthermore, modulation of apoptosis-related molecules and pathways may lead to the development of innovative therapies for conditions where apoptosis dysregulation plays a vital role [182,183].

### 4.1. Targeting Apoptosis for Enhanced Cancer Treatment

Significant efforts have been dedicated to the development of diverse and promising experimental anticancer drugs that can influence apoptotic pathways [184,185]. Given that apoptosis plays a dual role, researchers aim to target any defects or abnormalities along these pathways to restore apoptotic signaling and eradicate cancer cells. One key target for intervention is the anti-apoptotic Bcl-2 family of proteins. The overexpression of these pro-survival members is associated with aggressive cancer and chemoresistance, making them highly attractive therapeutic targets for pharmacological manipulation of cell death [186]. Studies have demonstrated that using small interfering RNAs (siRNAs) to inhibit these pro-survival Bcl-2 family members can induce apoptosis and lead to a reduction in tumor growth [187,188]. For example, specific downregulation of Mcl-1 through siRNA resulted in significant apoptosis of the leukemia cells (HL-60) in vitro [189]. Additionally, certain microRNAs, such as miR-195, miR-24-2, miR-365-2, miRNA-15a, and miRNA-16-1, have been found to regulate Bcl-2 expression, highlighting their therapeutic potential in this context [10,190,191,192]. Multiple drugs that are designed to target key components of apoptosis are in development phases, with some showing promising results in clinical phases [193], as shown in Table 1.

### 4.2. Strategies for Inducing Apoptosis

#### 4.2.1. Bcl2 Antisense

This approach involves downregulating Bcl-2 proteins by targeting their corresponding mRNA [194]. For instance, Oblimersen sodium (G3139, Genasense), an 18-antisense oligonucleotide, was the first agent targeting Bcl-2 mRNA to enter clinical trials [195,196,197]. Preclinical studies have shown that Oblimersen sodium reduces Bcl-2 expression, leading to cytotoxic and cytostatic effects in various cancer cells, including breast, ovarian, prostate, melanoma, non-small-cell lung carcinoma (NSCLC), bladder, and gastric cancers [198,199,200,201,202,203,204]. Clinical trials combining the antisense agent with cytotoxic anticancer drugs have also been pursued to enhance drug sensitivity in different cancer cells.

#### 4.2.2. Small Molecule Inhibitors of BCL-2 Proteins: These Inhibitors Can Be Classified into Two Groups

(a) Molecules affecting gene or protein expression

Certain components, such as members of the steroid/retinoid superfamily of ligand-activated transcription factors (SRTFs), offer potential targets for modulating *Bcl-2* and *Bcl-xL* genes [205,206]. Small molecule drugs that modulate the activity of retinoic acid receptors (RARs), retinoid X receptors (RXRs), PPAR, and vitamin D receptors (VDRs) have shown promise in downregulating Bcl-2 or Bcl-xL expression in various cell lines including IEC-6, K562, etc. [207,208,209,210,211]. For example, Troglitazone or Δ2-TG, a potent PPAR agonist, has been found to inhibit the anti-apoptotic functions of Bcl-xL and Bcl-2, triggering caspase-dependent apoptosis [212,213]. Other molecules, such as histone deacetylase inhibitors, synthetic cytotoxic retinoids, cyclin-dependent kinase inhibitors, and deubiquitinase inhibitors, downregulate RNA expression in human T lymphoma (DHL-4 and DHL-6) and hepatic (HCC) cell lines [214,215,216,217,218].

(b) Molecules targeting proteins

These small molecules directly bind to anti-apoptotic Bcl-2 proteins at their BH3-binding groove, mimicking the action of BH3 proteins [219]. Examples of such agents include gossypol, Obatoclax (GX15-070), ABT-737, and ABT-263 or navitoclax, which target different anti-apoptotic members of the Bcl-2 family, such as Bcl-2, Bcl-xL, Bcl-W, Mcl-1, Bcl-B, and Bfl-1, thereby exhibiting potential antitumor efficacy [220] Table 1.

**Table 1:** Trials (clinical and preclinical) of drugs targeted for cancer cells through apoptotic pathways.

Drugs	Mechanism/Target	Trial Phase	Type of Cancer	Trial ID and References
1. Targeting Anti-Apoptotic Bcl-2 Family Members Using Bcl-2 Antisense *				
Oblimersen Sodium (G3139, Genasense (trial name))	Inhibitor Bcl-2	Phase I		NTC00039117/(NCI)
		Phase I/II	Acute myeloid leukemia (combined with cytarabine and Daunorubicin)	NTC00055822/(NTC)
		Phase I/II	Advanced colorectal cancer (combined with chemotherapy)	NTC00064259/(NTC)
		Phase I/II	Advanced esophageal, gastroesophageal junction, and gastric cancer (combined with Cisplatin and Fluorouracil)	NCT00063934/(NTC)
		Phase II	Advanced breast cancer (combined with Doxorubicin and Docetaxel)	NCT00079131/(NCT)
		Phase II	Merkel cell carcinoma	NCT00049192/(NCT)
			Chronic myelogenous leukemia (combined with Imatinib Mesylate)	
		Phase II	B-cell Hodgkin lymphoma(combined with cisplatin and 5-fluorouracil)	NCT00054639/(NCT)
		Phase II	Chronic myelogenous leukemia (combined with Imatinib Mesylate)	NCT00049192/(NTC)
		Phase II	Small-cell lung cancer (combined with carboplatin/etoposide)	NCT00042978/(NTC)
		Phase III	Multiple myeloma and plasma cell neoplasms (with or without dexamethasone)	NCT00017602/(Genta Incorporated, Berkeley Heights, NJ, USA)
		Phase III	Chronic lymphocytic leukemia (CLL) (combined with Fludarabine and Cyclophosphamide) and myeloma (plus dacarbazine)	NCT00024440/(Genta Incorporated)
		Phase III		NCT00016263 and NCT00518895/(Genta Incorporated)
**2. Strategy for p53 Activation Apoptosis**				
(a) Small Molecule MDM2 Inhibitors **				
R7112 (Nultlin-3 analogs of cis imidazoline)	Preventing MDM2-P53 interaction	Phase I	Advanced solid tumors	NTC00559533 (Hoffmann-La Roche, Basel, Switzerland)
JNJ-26854165 (tryptamine derivative)	Preventing MDM2-P53 interaction	Phase I	Advanced-stage or refractory solid tumors	NTC00676910 (Johnson & Johnson Pharmaceutical Research & Development, L.L.C, Raritan, NJ, USA)
(b) Small Molecules Restoring Mutated p53 to Wild-Type p53 ***				
SCH529074	Reactivates mutant P53 and inhibits ubiquitination of P53 by MDM2	Preclinical	WiDr, DLD-1, KLE, H322, MCF7, SJSA, WS-1, and MB-468 cancer cells	[221]
MIRA-3	Reactivates mutant p53	Preclinical	Tumor cells	[222]
PK083	Reactivation of Y220C mutant p53	Preclinical	Tumor cells	[223]
PRIMA-1	Reactivates mutant p53	Preclinical	Tumor cells (combined treatment with Cisplatin)	[224]
APR-246	Reactivates mutant p53	Phase I	Malignancies or hormone-refractory prostate cancer	[225]
475P–A (ALRN-6924 and palbociclib)	Reactivate p53	Phase IIa	Inhibits AML cell growth and clonogenic capacity	[226]
**(c) p53 Vaccines #**				
Adjuvant dendritic cell (DC)	Modified/optimized HLA class I binding peptides for CTL	Phase I	Head and neck squamous cell carcinoma	[227]
Peptide-based vaccines	Facilitate the induction of antitumor immune responses, particularly antitumor CTL	Phase I/II	- Colorectal cancer	[228]
		Phase II	- Ovarian cancer	[229]
Dendritic-cell-based vaccine		Phase II	- Advanced (breast, cervical, colorectal, lung, ovarian, pancreatic)	NCT00019084
Recombinant virus		Phase I/II	- Breast cancer	[230]
		Phase I/II	- Colorectal cancer	[231]
		Phase I/II	- Small cell lung cancer	[232]
		Phase I	- Brain and central nervous system tumors	NCT00004080 (NCI, Bethesda, MD, USA)
**3. Antibodies Targeting TRAIL Receptors ##**				
Dulanermin	Targets TRAIL-R1 and TRAIL-R2	Phase I/II	- Non-Hodgkin’s lymphoma (combined with Rituximab)	NCT00400764 (Genetech, Inc., San Francisco, CA, USA)
Mepatumumab (HGS ETR1, TRM-1)	Targets TRAIL-R1	Phase II	Multiple myeloma (combined with Bortezomib)	NCT00315757 (Human Genome Science Inc., Rockville, MD, USA)
Lexatumumab (AMG 655)	Targets TRAIL-R2	Phase I	- Metastatic tumors	NCT00428272 (NCI)
Conatummab (HGS-ETR2)	Targets TRAIL-R2	Phase I/II	- Colon cancer, colorectal cancer, rectal cancer, metastatic colorectal cancer oncology (combined with panitumumab)	NCT00630786 (Agmen, Thousand Oaks, CA, USA)
Drozitumab	Targets TRAIL-R2	Phase Ib	- Metastatic colorectal cancer (mCRC) (combined with cetuximab + irinotecan or FOLFIRI ± bevacizumab)	[233]
Tigatuzumab	Targets TRAIL-R2	Phase II	- Breast cancer (combined with Abraxane)	NCT01307891 (University of Alabama at Birmingham, Birmingham, AL, USA)
LBY135	Targets TRAIL-R2	Phase I/II	- Solid tumor (with or without capecitabine)	[234]
**4. Dual Bcl-2 and Bcl-xl Inhibitor ###**				
**Navitoclax**	**Inhibits BIM and anti-apoptotic protein interactions**	**Phase I/II**	**CLL** **Advanced solid tumors** **Melanoma NSCLC**	NCT02079740 NCT02520778 NCT01989585 NCT02143401
APG-1252	Binds specifically to BCL-2 or BCL-XL’s hydrophobic pocket	Phase I/II	SCLC Advanced solid tumors	NCT03387332
**5. Selective BCl-2 Inhibitor †**				
Venetoclax	Selectively binds to BCL-2, displacing pro-apoptotic	Phase I-III	CLL	FDA approved
			NHL	
			AML	
S55746 (BCL201)	Externalization of phosphatidylserine, caspase-3 activation, and PARP cleavage	Phase I	CLL	NCT02920697 NCT02603445
			NHL	
			Multiple myeloma	
APG-2575	Activate the mitochondrial apoptotic pathway	Phase I	CLL	NCT03913949 NCT03537482
			NHL	
**6. BCl-xl Inhibitor ††**				
Mirzo-C	Conjugate of three; specifically the target of BCL-X_L_	Phase I	Solid tumors	NCT03595059
**7. MCL1 Inhibitor †††**				
AMG176	Binds to the BH3-binding groove of MCL-1	Phase I	AML NHL	NCT03797261 NCT02675452
			Multiple	
			Myeloma	
MIK665 (S64315)	Through induced loss of *NOXA*	Phase I	AML	NCT02992483 NCT02979366 NCT03672695
			NHL	
			Multiple myeloma	
AZD5991	Direct inhibitor of MCL-1	Phase I	NHL	NCT03218683
			AML	
			ALL	
			Multiple myeloma	
**8. Inhibitor of Apoptosis (IAP) and SMAC Mimetics ‡**				
LCL161	Activation of non-canonical NF-κB pathways and production of TNF-α	Phase I/II	Advanced solid tumors	NCT02649673 NCT03111992 NCT02098161
			Breast cancer	
			Colorectal	
			NSCLC	
			SCLC	
			Multiple myeloma	
			Polycythemia Vera	
			Myelofibrosis	
Birinapant (TL32711)	Binds IAPs, promotes their degradation	Phase I/II	Advanced solid tumors	NCT02587962 NCT03803774
			NHL	

* A dose of 3–7 mg/kg/day given as a continuous intravenous infusion; side effects mainly include neutropenia, hypokalemia, mucositis, fatigue, dizziness, thrombosis, and dehydration. ** A single oral dose of the compound at 200 mg/kg, toxic to normal tissues also. *** Dose depends on the stage and size of the tumor, mostly well tolerated. # Three to four doses at 3-week intervals, multiple routes, reported to be safe, yet survival needs to be analyzed. ## The dose depends on the type of cancer, given as intravenous/intradermal infusion, which causes infusion reactions as they are proteins. ### Given as a single intravenous agent or combination depending on the type and stage of cancer. Side effects include thrombocytopenia and gastrointestinal and hematological toxicities. † Same as above with neurotoxicity. †† Orally bioavailable, 150–325 mg/day, intermittently or continuously; common toxic effects included grade 1 or 2 anemia, infection, diarrhea, nausea, fatigue, thrombocytopenia, lymphocytopenia, and neutropenia. ††† Variable doses or can be given as a single drug, disrupt mitochondrial homeostasis, and potential cardiac toxicity. ‡ A dose of 45 mg weekly or depending on the type or stage of cancer, intravenous infusion, and manageable safety profile.

## 5. Some Potential Future Therapeutic Perspectives and Directions for Apoptosis

Apoptosis plays a crucial role in maintaining cellular integrity and eliminating damaged or abnormal cells, including cancer cells. Research is likely to continue in exploring ways to manipulate apoptosis for targeted cancer therapies. Understanding the intricate signaling pathways involved in apoptosis could lead to the development of drugs that specifically induce apoptosis in cancer cells while sparing healthy ones, resulting in more effective and less toxic treatments [235]. Neurodegenerative disorders, such as Alzheimer’s and Parkinson’s diseases, are characterized by the progressive loss of functional neurons. The dysregulation of apoptosis has been implicated in these conditions. Future research may focus on identifying ways to modulate apoptosis in the brain to prevent neuronal loss and potentially slow down the progression of these devastating diseases [236].

Apoptosis is also involved in the regulation of immune responses, including the elimination of self-reactive immune cells during development and the resolution of immune reactions. Further research into the apoptotic pathways in immune cells could lead to the development of therapies for autoimmune diseases by selectively inducing or inhibiting apoptosis in specific immune cell types [9,237]. The role of apoptosis in various cardiovascular diseases, including heart failure and atherosclerosis is also established. Future studies may explore the potential of apoptosis modulation to prevent cell death in cardiac tissues or to target specific cells involved in the progression of cardiovascular diseases [238,239]. Understanding apoptosis is crucial in the context of tissue regeneration and wound healing. Future research might focus on how apoptosis influences tissue remodeling and regeneration, to enhance regenerative approaches for various injuries and diseases [240,241,242,243]. Aging is associated with an imbalance between cell death and cell renewal processes, and apoptosis is a key component. Investigating the role of apoptosis in aging may lead to potential interventions to promote healthy aging and reduce age-related diseases [244].

## 6. Advanced Techniques in Apoptosis Research

As technology continues to advance, the development of innovative tools and techniques to study apoptosis at the molecular level with higher precision and resolution is ongoing. This could lead to new insights into the regulation and dysregulation of apoptosis in various contexts. Overall, the study of apoptosis holds immense promise for advancing our understanding of cellular biology and its implications in health and disease. Continued research in this field has the potential to pave the way for novel therapeutic strategies aimed at maintaining cellular equilibrium and promoting human health [245]. Regenerative medicine is an advanced technique for the regeneration of tissues and organs. Bone marrow mesenchymal stem cells (BMSCs) are one of the most common stem cells used for this therapy, with a promise to show good outcomes in degenerative diseases in humans and animals. BMSC transplantation is a promising regenerative therapy with a dismal survival rate due to the build-up of oxidative stress. Understanding the in-depth mechanism of apoptosis and its rate in BMSCs will pave the way for better therapeutic outcomes. Along with this, vascular smooth muscle cells are present in blood vessels and are responsible for the remodeling of blood vessels following injury. One recent therapeutic approach is the activation of intracellular signaling cascades governing cell proliferation and apoptosis. Exploring the intricate molecular mechanisms may amplify the effects of vessel remodeling and disease.

Treatment modalities targeting P53 and the pro-apoptotic BCL-2 homology 3 family elicit mitochondrial apoptosis when a patient is treated with radiation and chemotherapy. They have been reported to induce excessive apoptosis in cardiovascular and neurodegenerative diseases and ovarian cancer treatments [246,247,248].

Fluorescence resonance energy transfer (FRET) is used to detect apoptosis in single cells in real time in live animal models. Bioluminescent probes and nanoparticle-based assays like carbon dots (CDs) can be used for apoptosis detection in vitro. Other approaches include luminol electrochemiluminescence and immunolabeling technology using fluorescein, radioisotopes, and electron-dense substances. These innovative approaches have great potential for both in vitro and in vivo studies due to their increased sensitivity, time efficiency, specificity, and negligible cytotoxicity. Unfortunately, they still are less used in real applications as their optimization and improvement are still being performed in research studies [249,250].

## 7. Conclusions and Prospects

Research into apoptosis, an essential cellular activity, has discovered sophisticated molecular processes governing cell survival and death. Over the years, our understanding of apoptosis has grown substantially, embracing many types and pathways that coordinate a finely tuned equilibrium in multicellular organisms. A key role in interpreting the intricate signaling that controls apoptosis has been played by caspases, the proteolytic enzymes essential to the process. Apoptosis events are highly organized and entail a succession of synchronized processes, beginning with initiation signals and ending with the execution phase, in which the cell undergoes regulated breakdown. This planned cell death is required not just for normal growth and tissue homeostasis, but also for the elimination of damaged or possibly harmful cells. However, apoptotic dysregulation has been linked to a variety of diseases. In medical research, the ability to target apoptosis for the treatment of diseases linked to it has been identified as a potential target. Manipulation of apoptotic pathways has the potential to lead to the development of novel therapeutics for diseases characterized by either excessive or inadequate cell death. Dysregulation of apoptosis in diseases emphasizes the necessity of identifying key molecular factors that can be addressed for therapeutic intervention. Advancement in biological science is continuously investigating ways to manage apoptosis, either by promoting cell death in diseases with uncontrolled cell division or by blocking excessive apoptosis in cells that cannot be regenerated. This focused strategy tries to restore apoptosis’s delicate equilibrium, providing new hope for patients suffering from diseases in which apoptosis is a crucial component in pathogenesis. Apoptosis manipulation shows immense promise, but problems remain. It is critical to fine-tune treatment interventions to achieve specificity and minimal off-target effects. Understanding the interaction between distinct cell death pathways and developing more accurate molecular tools will help to refine apoptosis-targeted medicines. To conclude, the study of apoptosis has provided significant new understandings of the intricate workings of biological life and mortality. There is hope for alternative and successful treatments for a variety of illnesses as scientists continue to pore over the molecular details of apoptotic pathways and explore potential therapeutic applications and apoptosis manipulation. Unquestionably intriguing, the field of apoptosis study has enormous potential to transform medical problems in the future.

The discipline of apoptosis has broadened in knowledge and covers all the areas of life. Apoptosis has been researched in almost every area of biology. Despite the detailed understanding and an in-depth understanding of apoptosis at the molecular level, some areas remain unexplored, specifically in relation to the development of novel therapies. Understanding and revisiting the mechanisms of apoptotic signals and their transductions will pave the way for better therapeutic outcomes. This will help specifically eliminate the altered, transformed, and damaged cells while sparing the healthy lot. Also, this will protect and minimize the risk of damage to healthy tissues and prevent progressive degeneration and associated disorders. Further research is necessary to decipher the interconnectivity between signaling, cell cycle, and controlled proliferation for better drug targeting toward cancers and various pathogen-associated diseases to identify and correlate the intricate balance between cell survival and death.

## Data Availability

Not applicable.
